# Neurotrimin, a neural adhesion molecule, expression in early and advanced stage endometriosis

**DOI:** 10.61622/rbgo/2025rbgo57

**Published:** 2025-09-12

**Authors:** Derya Iliman, Cihan Kaya, Sibel Kuras, Alev Kural, Murat Ekin, Levent Yasar

**Affiliations:** 1 Obstetrics and Gynaecology Department Southmead Hospital North Bristol NHS Foundation Trust Bristol United Kingdom Obstetrics and Gynaecology Department, Southmead Hospital, North Bristol NHS Foundation Trust, Bristol, England, United Kingdom.; 2 Istanbul Aydin University Faculty of Medicine Department of Obstetrics and Gynecology Istanbul Turkey Istanbul Aydin University Faculty of Medicine Department of Obstetrics and Gynecology, Istanbul, Turkey.; 3 Department of Medical Biochemistry University of Health Sciences Hamidiye Faculty of Medicine Istanbul Turkey Department of Medical Biochemistry, University of Health Sciences, Hamidiye Faculty of Medicine, Istanbul, Turkey.; 4 Department of Biochemistry University of Health Sciences Bakirkoy Dr. Sadi Konuk Training and Research Hospital Istanbul Turkey Department of Biochemistry, University of Health Sciences, Bakirkoy Dr. Sadi Konuk Training and Research Hospital, Istanbul, Turkey.; 5 Department of Obstetrics and Gynaecology University of Health Sciences Bakirkoy Dr. Sadi Konuk Training and Research Hospital Istanbul Turkey Department of Obstetrics and Gynaecology, University of Health Sciences, Istanbul, Bakirkoy Dr. Sadi Konuk Training and Research Hospital, Bakirkoy, Istanbul, Turkey.

**Keywords:** Advanced endometriosis, Endometriosis, Endometriosis pathophysiology, Neurotrimin

## Abstract

**Objective:**

Endometriosis, a gynecological condition characterized by the presence of endometrial tissue outside the uterus, affects millions of women worldwide. This study aimed to investigate neurotrimin (NTM)’s expression, a glycerophosphatidylinositol-anchored neural adhesion molecule, in endometriotic lesions and eutopic endometrial tissue.

**Methods:**

In this laboratory based observational study NTM expression was measured in patients with early- and advanced-stage endometriosis and controls (patients without endometriosis who underwent gynecological surgery. Peritoneal endometriosis (peritoneum for controls) and eutopic endometrial tissue samples were collected from patients. Polymerase chain reaction and immunohistochemistry (IHC) were used to detect NTM in the tissue samples. Additionally, NTM levels in peripheral blood samples of all participants were measured using an enzyme-linked immunosorbent assay.

**Results:**

NTM mRNA and protein levels were significantly higher in the endometriotic foci of the stage 3–4 endometriosis group than in the control group (p<0.01). Stage 1–2 endometriotic foci showed significantly higher NTM IHC staining than the control group; however, no significant difference was found between the mRNA levels. Eutopic endometrial tissue from the stage 3–4 group had significantly higher NTM mRNA levels than the other groups. No significant difference was found between the control and stage 1–2 groups for eutopic tissue. Eutopic endometrial NTM IHC staining did not differ between groups. No significant difference was observed in peripheral blood NTM levels.

**Conclusion:**

This study found increased NTM expression, a neural adhesion molecule, especially in advanced endometriosis. The endometrial tissue of patients with early-stage endometriosis also showed increased NTM expression in ectopic locations but not in eutopic tissue.

## Introduction

Endometriosis, a debilitating disease affecting an estimated 10% of women worldwide,^(1)^ lacks a cure and relies on management through medical and surgical interventions. This translates to a significant number of women living with a condition that has limited response to current treatments. Understanding the pathophysiology of this condition is a crucial step towards better treatment and possibly a cure.^(2,3)^

Endometriosis is known to be dependent on steroid hormones, particularly estrogen. Estrogen levels are elevated in endometriosis due to upregulation of p450 aromatase expression. In addition, progesterone resistance causes a lack of induction of 17-B-Hydroxysteroid dehydrogenase type 2 (17 β-HSD2), which converts estradiol to the less potent estrone.^(4,5)^ Thus, estrogen accumulates in its most potent form owing to altered hormone regulation during endometriosis.^(6-8)^

Beyond hormonal and inflammatory factors, the neuronal effects of endometriosis have been investigated. Unlike healthy controls, the endometrial basal layer in patients with endometriosis exhibits multiple fine unmyelinated nerve fibers.^(9)^Moreover, a decrease in sympathetic nerve density and an increase in sensory nerve density have been observed in peritoneal endometriotic lesions compared with the peritoneum of healthy controls. Furthermore, after hormone treatment, a reduction in nerve density was observed in peritoneal endometriosis and eutopic endometrium.^(10)^ In adult rodents, sympathetic nerve degeneration occurs when estrogen levels peak in the cycle.^(11)^ Notably, these estrogen fluctuations lead to changes in sympathetic and sensory innervation, ultimately leading to endometriotic vaginal hyperalgesia in rats.^(12)^ Furthermore, a positive correlation has been established between pelvic pain and nerve fiber density in patients with endometriosis.^(13)^ This growing body of evidence suggests that estrogen-modulated changes in nerve density directly contribute to endometriosis-associated pain. Researchers are actively exploring various molecular pathways to elucidate the mechanisms by which estrogen causes an imbalance in nerve density.^(14)^

Neurotrimin (NTM), a glycophosphatidylinositol (GPI)-anchored neuronal adhesion molecule belonging to the immunoglobulin LSAMP, OBCAM, NTM (IgLON) family, is primarily found in neural tissue.^(15)^ In a previous study, it was observed that estrogen exerts its effects through NTM and brain-derived neurotrophic factor (BDNF). When NTM synthesis is blocked, sympathetic axon degeneration is prevented.^(16)^ Unlike BDNF, NTM promotes sensory neuron growth through homophilic interactions, leading to the degeneration of sympathetic neurons through heterophilic interactions.^(17)^ Sympathetic nerves are known to promote anti-inflammatory changes. In contrast, sensory nerves promote a pro-inflammatory environment.^(18,19)^ Notably, both neural and inflammatory changes occur simultaneously within endometriotic lesions, and estrogen appears to influence these changes directly.^(18,20)^ Therefore, excessive estrogen via NTM may be the first step toward inducing neuronal and inflammatory changes in endometriotic lesions.

Given that neurotrimin (NTM) plays a role in neural remodelling, and that estrogen modulates both NTM expression and neural plasticity, we hypothesize that aberrant estrogen-driven NTM expression may contribute to the degeneration of sympathetic nerves and the growth of sensory neurons, thereby facilitating the establishment and maintenance of endometriotic lesions. This study aimed to evaluate the levels of NTM expression in endometriotic foci and eutopic endometrium of patients with endometriosis. To our knowledge, this is the first study to investigate the role of neurotrimin (NTM) in estrogen-mediated neural remodelling in endometriosis, offering a novel perspective on the mechanisms underlying nerve subtype imbalance in this disease.

## Methods

We designed a laboratory based observational study to demonstrate NTM expression in endometriosis. Our study population included patients diagnosed with endometriosis who underwent surgery at a tertiary referral center between September 2020 and January 2022. All consecutive patients evaluated during the study were considered eligible unless they met any exclusion criteria. Eligible patients were included in the study after providing detailed medical information and written informed consent. Exclusion criteria included known cardiac, central/peripheral neural, autoimmune, muscle, or sensory disorders; leiomyoma; smoking; obesity and hormone treatment within the 3 months before surgery. The control group consisted of patients who underwent surgery at the same tertiary center for benign indications other than endometriosis or leiomyoma and who did not meet any exclusion criteria.

The study design employed three groups: Group 1 comprised patients who underwent laparoscopy for non-endometrioma benign cysts or diagnostic reasons; Group 2 included patients with stage 1–2 endometriosis according to the revised-American Society for Reproductive Medicine (rASRM) endometriosis severity scoring; and Group 3 comprised patients with stage 3–4 endometriosis based on rASRM endometriosis staging. The operating surgeon assigned patients to their respective groups. Criteria to be assigned in control group included no visible endometriosis during surgery as well as no suspicion of endometriosis symptoms in patient history. This measure taken to ensure no endometriosis in the control group. NTM expression and levels were investigated in the endometriosis groups using biopsies taken from endometriotic lesions on the peritoneal surface and endometrium. Preoperative venous blood samples were also collected from patients in the endometriosis groups. In the control group, these samples were investigated through biopsies obtained from the peritoneum and endometrium and preoperative venous blood samples. All surgeries were conducted within the first 5 days of the individual patient’s menstrual cycle.

Venous blood samples taken from individuals were centrifuged at 3000xg for 10 minutes. The supernatant was transferred to tubes and stored at -80°C until analysis on the day of the study. Frozen serum samples were thawed at room temperature before use. NTM levels in the samples were measured according to the manufacturer’s protocol using a commercially available ELISA kit (Human NTM ELISA Kit, 201-12-3033; Sunred, Shanghai, China). ELISA was performed at the Medical Biochemistry Research Laboratory of Health Sciences University, Turkey.

Tissue samples were pre-homogenized on ice, and the total RNA was then isolated using a Quick-RNA Miniprep Plus Kit (R1058; Zymo Research, Irvine, CA, USA) per the manufacturer’s instructions. To determine the tissue NTM gene expression, we equalized total RNA concentrations. Complementary DNA (cDNA) was synthesized from the equalized total RNA samples using a cDNA synthesis kit (SensiFAST cDNA Synthesis Kit; Cat. No: BIO-65054; Bioline, Memphis, TN, USA).

cDNA synthesis was carried out in a total volume of 20 µL containing 5 µL of total RNA, 4 µL of 5x TransAmp Buffer, 1 µL of reverse transcriptase enzyme, and 10 µL of RNase-free water. The reactions were subjected to the following thermal cycling conditions: 25°C for 10 minutes, 42°C for 15 minutes, 85°C for 5 minutes, and 4°C hold. For qRT-PCR, GAPDH was used as the reference gene for RNA expression detection. After cDNA synthesis, qRT-PCR analysis was performed with SensiFAST™ SYBR No-ROX kit. The qPCR reactions were carried out with a total volume of 20 µL containing 10 µL of 2x SensiFAST SYBR No-ROX Mix, 0.8 µL of each primer, 5 µL of cDNA, and 3.4 µL of RNase-free water. The following NTM gene primers were used: CCACACCAATGCCAGCATCATG (forward) and TGAGAAGCAGGTGCAAGACCAG (reverse). The qPCR reactions were subjected to hot start at 95°C for 2 minutes, followed by 40 cycles of denaturation at 95°C for 5 seconds, annealing at 54°C for the NTM gene and 58°C for the GAPDH gene for 30 seconds, and extension at 72°C for 20 seconds using the real-time detection system (CFX96 Touch; BioRad, Hercules, CA, USA). qRT-PCR was performed twice for each sample. The cycle threshold (Ct) was obtained as a qRT-PCR result for each repetition of each gene. The mean Ct value was calculated by averaging the two results. The expression of genes was quantified by measuring the Ct values and normalized using the 2-∆∆CT method relative to the GAPDH RNA.

All tissue samples were fixed in 10% neutral-buffered formaldehyde and embedded in paraffin. Paraffin slices were cleaned with xylene and rehydrated through a series of decreasing alcohol concentrations. Sections were then stained with hematoxylin and eosin (H&E) according to the laboratory protocol. For NTM, deparaffinization was performed with 1/10 ethylenediaminetetraacetic acid (EDTA) buffer (pH 8.0) (AP-9004-999; ThermoScientific, Waltham, MA, USA) PT Module (A80400012 Lab Vision). The tissues were treated with 3% hydrogen peroxide (TA-125-HP ThermoScientific) for 10 minutes to inactivate endogenous peroxidase activity. The tissues were washed with phosphate-buffered saline (PBS) and blocked with Protein Block (TA-125-PBQ ThermoScientific) for 10 minutes. The primary antibody, anti-neurotrophin antibody (orb9468 Biorbyt), was diluted 1:100 and incubated for 60 minutes. Further, the Quanto amplifier (TL-125-QPB ThermoScientific) was incubated for 20 minutes, followed by incubation with HRP Polymer Quanto (TL-125-QPH ThermoScientific) for 30 minutes. After each incubation step, cells were washed with PBS. To identify myeloperoxidase-positive cells, we performed staining using the DAB Chromogen. For background staining, Haematoxylin (HHS32 Sigma) was applied for 30 seconds. Furthermore, immunohistochemical staining was performed using a Shandon/Thermo Sequenza model IHC device (Sequenza Immunostaining Center Each 73300001 Shandon/Thermo). To quantify optical density, we captured x40 magnification photographs from systematically randomized regions selected from the tissue and measured using the ImageJ software. Fibrosis, cell and blood vessel counts, and other pathological findings were measured using Cavalieri’s principle on histological sections. For immunohistochemical analysis, the H score was calculated as follows: the presence of staining, staining intensity, and area of staining were scored on a scale of 0 to 4 (0 = no staining, 4 = intense staining). The average score was calculated for all the sections and multiplied by 100 to obtain an H score ranging from 0 to 400.

**Figure 1:**
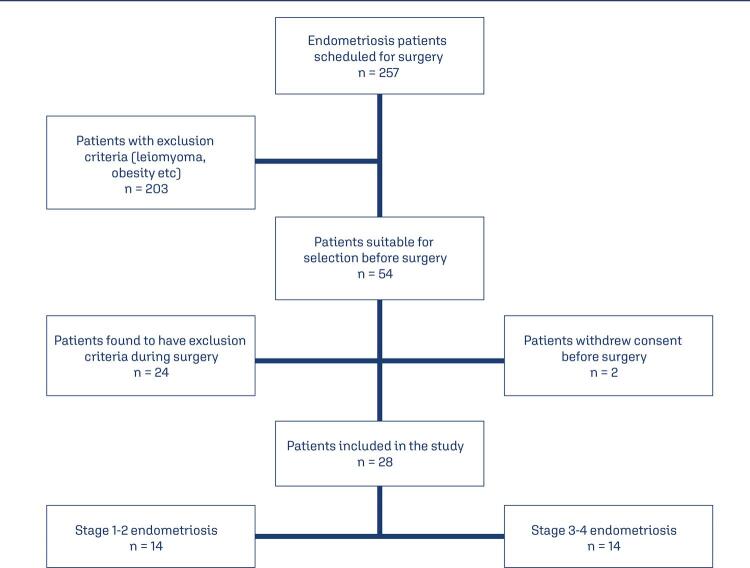
Patient selection flowchart for study groups

**Figure 2:**
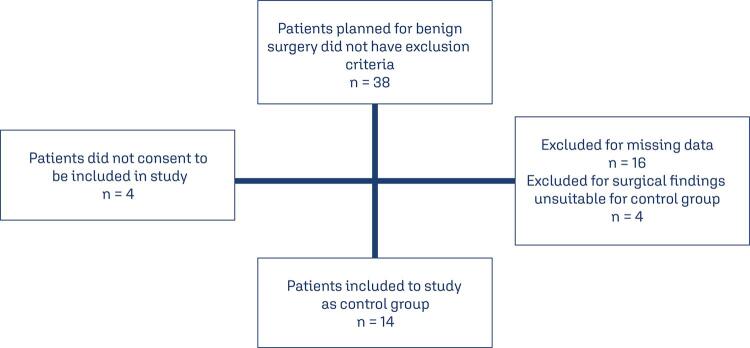
Patient selection flowchart for control group

A power analysis could not be performed due to the lack of previous studies on this topic. We enrolled 14 patients in each group and performed a post hoc analysis. We planned to enroll all suitable patients within 2-year period in our hospital. Patient selection flowchart for study and control groups are demonstrated in figure 1 and 2.

SPSS 25.0 and Modeler 18.0 software (IBM Corporation, Armonk, NY, USA) were used to analyze variables. Normal distribution of the data was assessed using the Shapiro–Wilk test, whereas variance homogeneity was evaluated using the Levene test. For group comparisons based on age and H-score, parametric tests such as one-way ANOVA (Robust Test: Brown-Forsythe) were used, followed by Tukey’s HSD test for post hoc analysis. For the comparison of groups based on the serum NTM fold change (for endometriosis or peritoneal samples and endometrium samples) and H score, non-parametric methods, such as the Kruskal–Wallis H test, were used, and Dunn’s test was used for post hoc analysis. To identify the most significant variables and for prediction purposes, several supervised machine learning methods were utilized, including logistic regression, discriminant analysis, support vector machine, random forest, K-nearest neighbor algorithm, simple (native) Bayes Classification, C5 algorithm from decision trees, and neural networks (Multilayer Perceptron-Radial Basis). Among these methods, the most successful model, the C5 algorithm (default), was used for the results presentation. Quantitative variables were expressed as mean (standard deviation), mean (standard deviation) (minimum–maximum), and median (minimum–maximum), whereas categorical variables were presented as n (%). Variables were examined at a 95% confidence level, and a p-value less than 0.05 was considered statistically significant.

Ethical approval for the study (approval number: 2020/374) was obtained from the local ethics committee.

## Results

**Figure 3:**
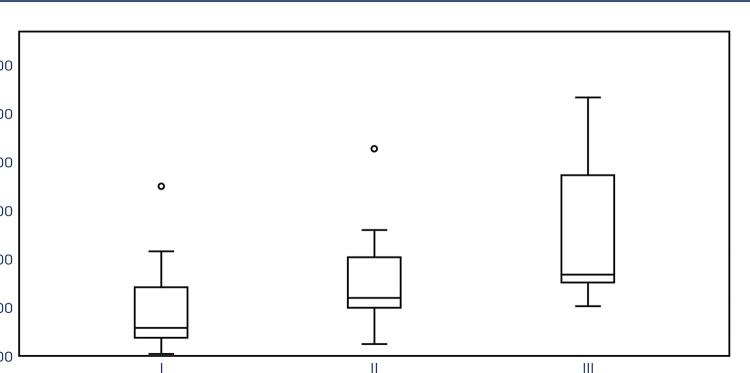
Peritoneal nodule or tissue PCR analysis graphic representation. Results presented as median (min-max) fold change. Increased scoring of stage 3-4 endometriotic nodules 3.33 (2.03-10.63) compared with control group 1.11 (0.06-6.96) and p=0.002. Increased mRNA levels demonstrated from group 1 (control) to group 2 and 3. However, statistical significance only achieved between control and stage 3-4 endometriosis groups I: Control II: Stage 1–2 endometriosis III: Stage 3–4 endometriosis

**Figure 4:**
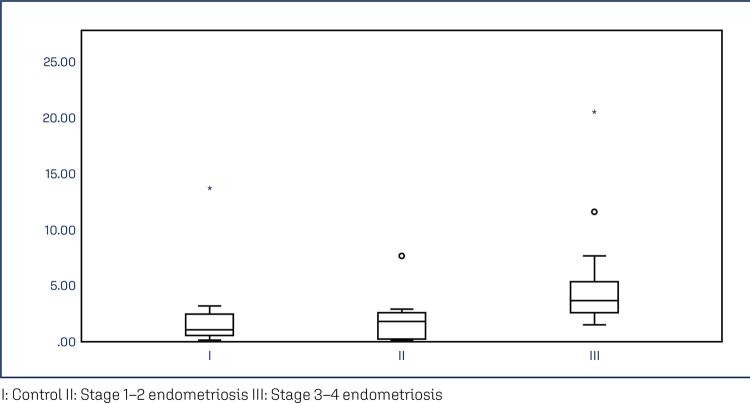
Eutopic endometrium PCR analysis graphic representation of the results. Results presented as median (min-max) fold change. Stage 3-4 endometriosis mRNA levels 3.63 (1.53-20.39) increased from control 1.07 (0.13-13.64) p=0.005 and stage 1-2 endometriosis 1.80 (0.08-7.67) p=0.016 A: Control B: Stage 1-2 endometriosis C: Stage 3-4 Endometriosis IHC: immunohistochemistry. Blue dyed neurotrimin increase on IHC from control to advanced stage endometriosis.

The study groups did not differ significantly in terms of age and BMI. The mean age was 39.93±4.21 in the control group, 34.93±5.65 in the stage 1–2 group, and 38.14±7.36 in the stage 3–4 group. Table 1 shows the comparisons and p-values for all variables. PCR analysis demonstrated that NTM mRNA expression in the endometriotic nodules was significantly higher than that in the control group (p<0.01). While median expression levels gradually increased from the control to the stage 1–2 and stage 3–4 groups, a statistically significant difference was observed only between the control and stage 3–4 groups (Figure 3). Additionally, PCR analysis revealed significantly higher levels of NTM mRNA expression in the eutopic endometrium of the stage 3–4 group compared with the other groups (Figure 4).

**Table 1:** Comparison of variables among groups

Variables	Control (1)	Stage 1-2 (2)	Stage 3-4 (3)	p-value
	Mean ± SD or Median (min-max)	Mean ± SD or Median (min-max)	Mean ± SD or Median (min-max)	
Age	39.93±4.21 (32-45)	34.93±5.65 (27-44)	38.14±7.36 (24-50)	0.090 ^x^
BMI	25.87±2.76	26.15±2.95	25.17±2.34	0.614 ^x^
Serum NTM	359.74 (85.65-956.09)	492.10 (35.34-5956.37)	476.24 (148.59-3808.69)	0.152 ^y^
Fold Change Endometrium^a^	1.07 (0.13-13.64)	1.80 (0.08-7.67)	3.65 (1.53-20.39)	0.001 ^y^
Fold Change Peritoneum or nodule^b^	1.11 (0.06-6.96)	2.41 (0.46-8.51)	3.33 (2.03-10.63)	0.002 ^y^
H Score Endometrium	130.16±74.91	151.56±68.18	134.11±67.43	0.694 ^x^
H score Peritoneum or nodule^c^	127.75 (77.80-233.30)	272.20 (122.20-355.50)	288.85 (111.10-388.90)	<0.001 ^y^

Control / Stage 3-4 endometriosis p:0.005

Control / Stage 1-2 endometriosis p:0.999

Stage 1-2 / Stage 3-4 endometriosis p: 0.016

^b^Between group comparisons for endometriotic nodule or peritoneal sample mRNA fold change

Control / Stage 3-4 endometriosis p:0.002

Control / Stage 1-2 endometriosis p:0.253

Stage 1-2 / Stage 3-4 endometriosis p: 0.253

^c^Between group comparisons for endometriotic nodule or peritoneal sample H scores

Control / Stage 3-4 endometriosis p:0.001

Control / Stage 1-2 endometriosis p:0.002

Stage 1-2 / Stage 3-4 endometriosis p: 0.999

^x^ One Way ANOVA (Brown-Forsythe) Test; Post Hoc Test: Tukey HSD Test,

^y^ Kruskal Wallis Test (Monte Carlo), Post Hoc Test: Dunn’s Test,

SD.: Standard Deviation

I: Control II: Stage 1–2 endometriosis III: Stage 3–4 endometriosis

**Figure 5:**
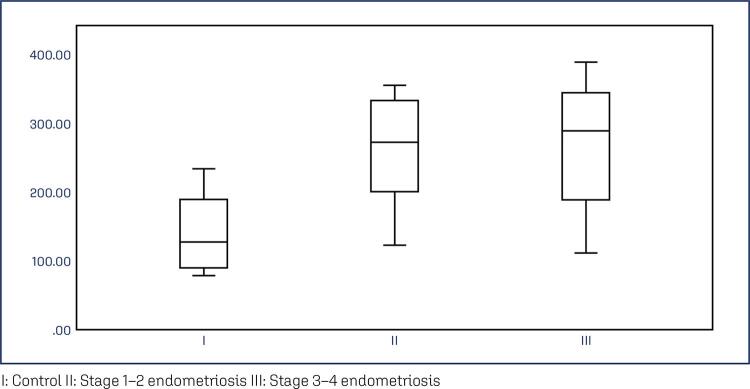
Peritoneal nodule or tissue IHC analysis graphic representation. Demonstrating increased staining of the endometriosis stage 3-4 of 288.85 (111.10-388.90) and stage 1-2 of 272.20 (122.20-355.50) from control group 127.75 (77.80-233.30) , p=0.001 and 0.002 ,respectively

**Figure 6:**
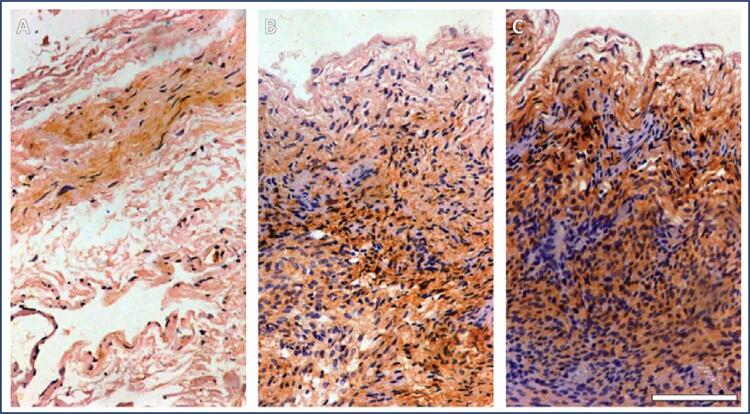
Peritoneal IHC. Scale:100 micron I: Control II: Stage 1–2 endometriosis III: Stage 3–4 endometriosis

Immunohistochemical staining was performed to assess the transcription levels of NTM proteins. Staining intensity for NTM protein levels in stage 1–2 and stage 3–4 endometriotic nodules was significantly higher than that in the control group (Figure 5). However, all groups had similar NTM protein levels in the eutopic endometrium. In addition, we conducted an ELISA to determine the NTM levels in the peripheral blood. Although NTM levels increased in patients with endometriosis, the results were not statistically significant (Figure 6).

## Discussion

Our study demonstrated elevated NTM RNA levels in both endometriotic nodules and eutopic endometrium of patients with advanced-stage endometriosis compared with healthy controls. This finding is in keeping with our hypothesis, as eutopic endometrium of patients with endometriosis shows certain alterations compared to healthy controls.^(18,21)^ One such difference is altered steroid hormone levels, specifically increased estrogen and progesterone resistance, in the eutopic endometrium.^(5,19)^ Increased NTM mRNA levels in the eutopic endometrium of the advanced stage endometriosis patients, is likely related to altered hormonal microenvironment. In a study by Parikh et al., leiomyoma cells were shown to have increased NTM production compared with normal myometrial cells. Furthermore, treatment of cells with 17-β -Estradiol up-regulated NTM protein expression in these cells.^(22)^ In another study, Krizsan-Agbas et al. demonstrated an increase in NTM mRNA levels in rat myometrium 6 hours after 17- β-Estradiol injection, with a return to baseline at 24 hours, further supporting the role of estrogen in NTM regulation.^(16)^These findings suggest that a high estrogen environment may enhance NTM transcription in the myometrium. Similarly, our study aimed to assess NTM mRNA and protein levels in both eutopic and ectopic endometrium of patients with endometriosis. Our results revealed increased NTM protein production only in the ectopic endometrium, while NTM mRNA levels were significantly elevated in both the ectopic and eutopic endometrium. This indicates that under eutopic conditions, NTM mRNA is not translated into the corresponding protein. The discrepancy between NTM mRNA and protein levels in the eutopic endometrium may be due to changes in epigenetic mechanisms, which lead to increased NTM protein production exclusively in the ectopic endometrium. This suggests that additional factors, likely inflammatory mediators, play a role in promoting NTM protein production in ectopic conditions. Endometriotic lesions are known to have elevated levels of inflammatory mediators, as well as alterations in neural and hormonal factors.^(23)^ One such change is an increase in neurotrophins, such as nerve growth factor (NGF), in the peritoneal fluid of patients with endometriosis.^(24)^

NGF acts as a mediator of inflammation by stimulating the release of neuroactive cytokines by inflammatory cells.^(25)^ Both sympathetic and sensory nerve fibers influence immune cells by locally releasing neurotransmitters.^(18)^ While the sensory nervous system plays a pro-inflammatory role through its mediators, the sympathetic nervous system exerts pro- and anti-inflammatory effects, depending on the inflammation stage.^(26,27)^ These findings highlight the connection between inflammatory and neural changes in endometriosis, mediated by NGF and other neurotrophic factors. Initially, the ability of estrogen to reduce sympathetic nerve density was attributed to NGF. However, it has been shown that NGF levels do not correlate with nerve density and that estrogen has a selective effect on reducing sympathetic density while sensory nerves increase.^(28)^ This selective effect was determined by NTM, and estrogen’s ability was impaired following NTM downregulation.^(16)^ Furthermore, alterations in nerve density have been linked to pain symptoms in endometriosis. In one study, patients who reported moderate-to-severe pelvic pain or dysmenorrhea had more nerve fibers associated with endometriosis, while those with reduced pain symptoms had fewer nerve fibers associated with endometriosis.^(13)^

Notably, NTM gene expression in the eutopic endometrium of patients with early-stage endometriosis was similar to that in the control group and significantly lower than that in the advanced-stage group. This finding is challenging to interpret, considering that few studies have investigated the pathophysiology of endometriosis at different stages. One study compared healthy control endometrium, eutopic endometrium of patients with endometriosis, and ectopic endometrium, demonstrating that miR-182, a micro-RNA, inhibits endometrial stromal cell proliferation, invasion, and inflammation in a dose-dependent manner by deactivating the NF-kβ signaling pathway.^(29)^ They reported decreased miR-182 levels from healthy to eutopic and ectopic endometrial tissues. Another study showed that increased miR-182 expression downregulated NTM and FGFR9 production after sciatic nerve injury and decreased Schwann cell migration and proliferation.^(30)^ Therefore, although these studies did not specifically consider different stages of endometriosis, they suggest that variations in factors such as miR-182 expression could contribute to differences in NTM production. It is plausible that the eutopic endometrium of patients with early-stage endometriosis may be more similar to that of healthy controls, while patients with advanced-stage endometriosis may exhibit further alterations in epigenetic factors, such as decreased miR-182 expression, leading to increased NTM production.^(31)^ However, further studies are needed to examine this potential association across different stages of endometriosis.

Our study observed increased NTM mRNA levels in endometriotic lesions, with levels rising from the healthy control peritoneum to stages 1–2 and 3–4. Statistically significant differences were found only between the control and stage 3–4 groups. Similarly, NTM protein levels were significantly lower in the peritoneum of the control group compared to all other groups. However, no significant difference in NTM protein levels was observed between the early and advanced-stage endometriosis groups, despite a median increase from 2.41 to 3.33. This increase, while notable, did not reach statistical significance, possibly due to variations in steroid hormone levels among patients. As previously mentioned, estrogen stimulates NTM mRNA levels within 6–24 hours, while NTM protein levels increase at both 6- and 24-hours following estrogen injection.^(16)^ Although this was observed in myometrial tissue, it demonstrates the effect of estrogen on both NTM mRNA and protein levels. Similarly, in our study, it is plausible that NTM protein levels were higher in endometriotic nodules regardless of hormone status, while NTM mRNA levels returned to baseline. This hypothesis, however, could be challenged by previous studies that observed sustained elevated estrogen levels in endometriotic tissue, independent of systemic estrogen levels.^(32)^ Nonetheless, the capacity for local estrogen production in early versus advanced-stage endometriotic nodules may differ. Such changes in local estrogen levels could help explain the observed differences in mRNA and protein levels between our study groups.

Our study suggests that NTM mRNA levels are increased in patients with advanced-stage endometriosis; however, a minimal amount of this mRNA is translated to produce protein levels in eutopic environments. The corresponding protein levels can be observed when endometrial cells are in ectopic tissues. Furthermore, patients with early-stage endometriosis remained between healthy controls and those with advanced-stage endometriosis. Their mRNA levels are much closer to those of healthy controls under eutopic conditions; however, once the cells are ectopic, both NTM mRNA and protein levels become closer to advanced-stage levels. To date, endometriosis staging has been a subject of debate, as progression from early to advanced stages has not been directly observed, and disease behavior is not predictable. The endometriotic disease theory suggests using the term endometriosis to describe subtle endometriotic cells usually seen in asymptomatic or early-stage women and endometriotic disease terms for advanced, symptomatic, cystic, deep, and typical lesions.^(33-35)^ Our findings in this study, particularly early- stage endometriosis group findings, are in keeping with this clinical variance. Molecular differences between early- and advanced-stage endometriosis may underlie the observed clinical variability and could help explain why a linear progression from early to advanced disease is not consistently observed.

Our study employed ELISA to test NTM levels in peripheral blood to determine its potential as a diagnostic marker. However, the NTM ELISA results were similar across all groups, likely due to the widespread distribution of NTM, especially in neural tissue.^(36,37)^

A strength of this study was the inclusion of patients in both early and advanced stages. The advanced-stage group consistently showed significantly increased NTM levels compared to healthy controls. However, the stage 1–2 group results were closer to the advanced stage in endometriotic lesions, while the eutopic endometrium results were more similar to those of the healthy controls. This aligns with existing literature suggesting potential variations in pathological changes between different endometriosis stages.^(38)^ Additionally, we used qPCR and immunohistochemistry to observe corresponding mRNA and protein levels.

A limitation of this study is the lack of pain scores.

## Conclusion

Our findings demonstrate increased NTM levels, which provide a connection between hormonal and neuronal changes in endometriosis. To our knowledge, this is the first study to explore the relationship between endometriosis and NTM levels. Further studies are therefore needed to investigate the functional impact of these changes, especially on pain pathophysiology. Investigating the pathways underlying pain symptoms in endometriosis patients may pave the way for the development of novel treatments or preventive strategies. In particular, we believe that pathways linking hormonal and neuronal changes could offer valuable insights for future research aimed at better controlling pain symptoms. Notably, including various stages of endometriosis is crucial, as early and advanced stages may exhibit significant differences in disease behavior.

## References

[1] Shafrir AL, Farland LV, Shah DK, Harris HR, Kvaskoff M, Zondervan K (2018). Risk for and consequences of endometriosis: a critical epidemiologic review. Best Pract Res Clin Obstet Gynaecol.

[2] Burney RO, Giudice LC (2012). Pathogenesis and pathophysiology of endometriosis. Fertil Steril.

[3] Cardoso JV, Perini JA, Machado DE, Pinto R, Medeiros R (2020). Systematic review of genome-wide association studies on susceptibility to endometriosis. Eur J Obstet Gynecol Reprod Biol.

[4] Noble LS, Simpson ER, Johns A, Bulun SE (1996). Aromatase expression in endometriosis. J Clin Endocrinol Metab.

[5] Zeitoun K, Takayama K, Sasano H, Suzuki T, Moghrabi N, Andersson S (1998). Deficient 17beta-hydroxysteroid dehydrogenase type 2 expression in endometriosis: failure to metabolize 17beta-estradiol. J Clin Endocrinol Metab.

[6] Zondervan KT, Becker CM, Koga K, Missmer SA, Taylor RN, Viganò P (2018). Endometriosis. Nat Rev Dis Primers.

[7] Noble LS, Takayama K, Zeitoun KM, Putman JM, Johns DA, Hinshelwood MM (1997). Prostaglandin E2 stimulates aromatase expression in endometriosis-derived stromal cells. J Clin Endocrinol Metab.

[8] Saunders PT, Horne AW (2021). Endometriosis: etiology, pathobiology, and therapeutic prospects. Cell.

[9] Tokushige N, Markham R, Russell P, Fraser IS (2006). High density of small nerve fibres in the functional layer of the endometrium in women with endometriosis. Hum Reprod.

[10] Arnold J, Barcena de Arellano ML, Ru¨ster C, Vercellino GF, Chiantera V, Schneider A (2012). Imbalance between sympathetic and sensory innervation in peritoneal endometriosis. Brain Behav Immun.

[11] Zoubina EV, Smith PG (2000). Axonal degeneration and regeneration in rat uterus during the estrous cycle. Auton Neurosci.

[12] Zhang G, Dmitrieva N, Liu Y, McGinty KA, Berkley KJ (2008). Endometriosis as a neurovascular condition: estrous variations in innervation, vascularization, and growth factor content of ectopic endometrial cysts in the rat. Am J Physiol Regul Integr Comp Physiol.

[13] Mechsner S, Kaiser A, Kopf A, Gericke C, Ebert A, Bartley J (2009). A pilot study to evaluate the clinical relevance of endometriosis-associated nerve fibers in peritoneal endometriotic lesions. Fertil Steril.

[14] Liang Y, Yao S (2016). Potential role of estrogen in maintaining the imbalanced sympathetic and sensory innervation in endometriosis. Mol Cell Endocrinol.

[15] Grijalva I, Li X, Marcillo A, Salzer JL, Levi AD (2006). Expression of neurotrimin in the normal and injured adult human spinal cord. Spinal Cord.

[16] Krizsan-Agbas D, Pedchenko T, Smith PG (2008). Neurotrimin is an estrogen-regulated determinant of peripheral sympathetic innervation. J Neurosci Res.

[17] Gil OD, Zanazzi G, Struyk AF, Salzer JL (1998). Neurotrimin mediates bifunctional effects on neurite outgrowth via homophilic and heterophilic interactions. J Neurosci.

[18] Straub RH (2007). Autoimmune disease and innervation. Brain Behav Immun.

[19] Cutolo M, Capellino S, Sulli A, Serioli B, Secchi ME, Villaggio B (2006). Estrogens and autoimmune diseases. Ann N Y Acad Sci.

[20] Zoubina EV, Mize AL, Alper RH, Smith PG (2001). Acute and chronic estrogen supplementation decreases uterine sympathetic innervation in ovariectomized adult virgin rats. Histol Histopathol.

[21] May KE, Villar J, Kirtley S, Kennedy SH, Becker CM (2011). Endometrial alterations in endometriosis: a systematic review of putative biomarkers. Hum Reprod Update.

[22] Parikh TP, Malik M, Britten J, Aly JM, Pilgrim J, Catherino WH (2020). Steroid hormones and hormone antagonists regulate the neural marker neurotrimin in uterine leiomyoma. Fertil Steril.

[23] Symons LK, Miller JE, Kay VR, Marks RM, Liblik K, Koti M (2018). The immunopathophysiology of endometriosis. Trends Mol Med.

[24] Anaf V, Simon P, El Nakadi I, Fayt I, Simonart T, Buxant F (2002). Hyperalgesia, nerve infiltration and nerve growth factor expression in deep adenomyotic nodules, peritoneal and ovarian endometriosis. Hum Reprod.

[25] Treede RD, Meyer RA, Raja SN, Campbell JN (1992). Peripheral and central mechanisms of cutaneous hyperalgesia. Prog Neurobiol.

[26] Härle P, Möbius D, Carr DJ, Schölmerich J, Straub RH (2005). An opposing time-dependent immune-modulating effect of the sympathetic nervous system conferred by altering the cytokine profile in the local lymph nodes and spleen of mice with type II collagen-induced arthritis. Arthritis Rheum.

[27] Karagiannides I, Pothoulakis C (2009). Substance P, obesity, and gut inflammation. Curr Opin Endocrinol Diabetes Obes.

[28] Brauer MM, Smith PG (2015). Estrogen and female reproductive tract innervation: cellular and molecular mechanisms of autonomic neuroplasticity. Auton Neurosci.

[29] Wu M, Zhang Y (2021). MiR-182 inhibits proliferation, migration, invasion and inflammation of endometrial stromal cells through deactivation of NF-?B signaling pathway in endometriosis. Mol Cell Biochem.

[30] Yu B, Qian T, Wang Y, Zhou S, Ding G, Ding F (2012). miR-182 inhibits Schwann cell proliferation and migration by targeting FGF9 and NTM, respectively at an early stage following sciatic nerve injury. Nucleic Acids Res.

[31] Gordts S, Koninckx P, Brosens I (2017). Pathogenesis of deep endometriosis. Fertil Steril.

[32] Chantalat E, Valera MC, Vaysse C, Noirrit E, Rusidze M, Weyl A (2020). Estrogen receptors and endometriosis. Int J Mol Sci.

[33] Koninckx PR, Donnez J, Brosens I (2016). Microscopic endometriosis: impact on our understanding of the disease and its surgery. Fertil Steril.

[34] Koninckx PR (1994). Is mild endometriosis a condition occurring intermittently in all women?. Hum Reprod.

[35] Koninckx PR, Ussia A, Adamyan L, Wattiez A, Gomel V, Martin DC (2019). Pathogenesis of endometriosis: the genetic/epigenetic theory. Fertil Steril.

[36] Struyk AF, Canoll PD, Wolfgang MJ, Rosen CL, D'Eustachio P, Salzer JL (1995). Cloning of neurotrimin defines a new subfamily of differentially expressed neural cell adhesion molecules. J Neurosci.

[37] Chen S, Gil O, Ren YQ, Zanazzi G, Salzer JL, Hillman DE (2001). Neurotrimin expression during cerebellar development suggests roles in axon fasciculation and synaptogenesis. J Neurocytol.

[38] Poli-Neto OB, Meola J, Rosa-E-Silva JC, Tiezzi D (2020). Transcriptome meta-analysis reveals differences of immune profile between eutopic endometrium from stage I-II and III-IV endometriosis independently of hormonal milieu. Sci Rep.

